# Lipids of lung and lung fat emboli of the toothed whales (Odontoceti)

**DOI:** 10.1038/s41598-020-71658-8

**Published:** 2020-09-08

**Authors:** Marina Arregui, Hillary Lane Glandon, Yara Bernaldo de Quirós, Idaira Felipe-Jiménez, Francesco Consoli, María José Caballero, Heather N. Koopman, Antonio Fernández

**Affiliations:** 1 Atlantic Cetacean Research Center, Institute of Animal Health (IUSA), Veterinary School, University of Las Palmas de Gran Canaria (ULPGC), Trasmontaña, s/n, 35413 Arucas, Las Palmas Spain; 2 Department of Biology and Marine Biology, University of North Carolina Wilmington (UNCW), Wilmington, NC 28403 USA; 3 Department of Neuroscience, Imaging and Clinical Sciences, University G. D’Annunzio, 66100 Chieti, Italy

**Keywords:** Lipids, Zoology, Pathogenesis

## Abstract

Lipids are biomolecules present in all living organisms that, apart from their physiological functions, can be involved in different pathologies. One of these pathologies is fat embolism, which has been described histologically in the lung of cetaceans in association with ship strikes and with gas and fat embolic syndrome. To assess pathological lung lipid composition, previous knowledge of healthy lung tissue lipid composition is essential; however, these studies are extremely scarce in cetaceans. In the present study we aimed first, to characterize the lipids ordinarily present in the lung tissue of seven cetacean species; and second, to better understand the etiopathogenesis of fat embolism by comparing the lipid composition of lungs positive for fat emboli, and those negative for emboli in *Physeter macrocephalus* and *Ziphius cavirostris* (two species in which fat emboli have been described). Results showed that lipid content and lipid classes did not differ among species or diving profiles. In contrast, fatty acid composition was significantly different between species, with C16:0 and C18:1ω9 explaining most of the differences. This baseline knowledge of healthy lung tissue lipid composition will be extremely useful in future studies assessing lung pathologies involving lipids. Concerning fat embolism, non-significant differences could be established between lipid content, lipid classes, and fatty acid composition. However, an unidentified peak was only found in the chromatogram for the two struck whales and merits further investigation.

## Introduction

Lipids are biomolecules present in all living organisms playing structural, metabolic, and endocrine roles. They are classified into two major groups: Polar (glycerophosphatides and sphingosylphosphatides), and nonpolar lipids (acylgycerols, waxes, sterols such as cholesterol, sterol esters, and free fatty acids)^1^.

Common lipids are present across the animal kingdom; however, there is also high diversity present in certain lipid types, mainly to meet specialized functions^1^. As an example of this, toothed whales (odontocete cetaceans) are the only mammals that deposit waxes within adipose tissues^2–7^. Blubber is a specialized hypodermis that constitutes the primary storage for metabolic energy in cetaceans^8^. Although in most species it is composed of triacylglycerols, it is dominated by waxes in the deepest diving toothed whales (i.e., families *Ziphiidae*, *Kogiidae*, and *Physeteridae*)^7,9,10^. All toothed whales have another set of specialized adipose depots called acoustic fats (melon in the forehead region and mandibular fats in and around the lower jaws), which are cranial fat bodies that participate in the transmission and reception of the sound^11,12^. The acoustic fats are composed of endogenously synthesized waxes and triacylglycerols, usually containing branched fatty acids and fatty alcohols^3,4,7,9,13^.

Apart from their physiological roles, lipids can also be involved in a great number of pathologies. One of them is fat embolism, which has been extensively described in human and animal science mainly associated with traumatic processes^14–17^, and it is defined as the mechanical obstruction of the lumen of blood vessels, mainly in the lung microvasculature, by circulating fat particles^17^.

As in other species, fat embolism in cetaceans is not a physiological or common finding in their lungs^18^ but related to various pathological entities as ship strikes, intra/interspecific traumatic interactions, or decompression-like sickness^18–22^. In traumatic cases (e.g., ship strikes and intra/interspecific interactions), the origin of fat emboli is thought to be bone marrow from fractured bones or from damaged soft tissues entering the bloodstream through torn venules^17,23^, as it has already been demonstrated in other species^24–28^.

Fat embolism has been described in combination with gas embolism (decompression-like sickness) in deep-diving species from the family *Ziphiidae* (beaked whales) that stranded in temporal and spatial association with naval exercises^21,29^. In this case, the origin of fat emboli remains unclear, although the most supported hypothesis postulates that gas bubble formation within the tissue may disrupt lipid-rich tissue, causing the entry of fat emboli in the bloodstream^21^. This hypothesis is supported by the fact that nitrogen is five times more soluble in lipids than in water^30,31^ and that in vivo formation of gas bubbles has been reported in the abdominal adipose tissue of mice under hyperbaric treatments^32^. Therefore, lipid-rich tissues can act as a nitrogen sink. Moreover, nitrogen solubility has been shown to be higher in blubber containing wax esters (i.e., beaked whales), than in blubber without^33^. Also, larger concentrations of shorter, branched-chain fatty acids and fatty alcohols (found in the acoustic fat bodies), together with an increased WE content, appear to increase nitrogen solubility^34^. All that was mentioned allows hypothesizing that waxes may have a crucial role in the genesis of fat emboli in cetaceans suffering decompression-like sickness.

To assess a pathological lipid composition in cetacean lungs and be able to identify fat emboli lipid composition accurately, prior knowledge of the typical lipid composition of these species' lung tissue is essential. However, data on lung tissue lipid composition in cetaceans are very limited. To our knowledge, lung tissue lipid composition has only been determined in a few cetacean species: *Stenella coeruleoalba* (striped dolphin)^35–37^ and *Phocoena phocoena* (harbour porpoises)^38^. In contrast, most studies have focused on the lipid composition of lung surfactant in cetaceans^39–42^ due to its primary physiological function involving alveolar stability to prevent lung collapse in air-breathing vertebrates^43–45^. The study of lung tissue composition instead of surfactant lipid composition is required for fat emboli assessment, as surfactant studies are performed based on pulmonary lavage while fat emboli are located within lung tissue vessels. Moreover, it would be interesting to assess if lung tissue lipid composition varies among distinct diving profiles (deep vs. shallow divers), considering the differences in nitrogen gas exposure likely experienced by deep versus shallow divers.

To provide these much-needed baseline data, the objectives of the present study were (1) to determine regular lung tissue lipid composition (lipid content, lipid class, and fatty acid composition) of cetacean species with distinct diving profiles (i.e., deep vs. shallow); and (2) to better understand the pathogenesis of fat embolism by determining the lipid composition of lungs known to be positive for fat emboli, and comparing those with lungs negative for emboli. In addition to determining the major lipid classes in the lungs, we also examined the polar lipid components in more detail as they are the major lipids in the lungs.

## Material and methods

### Sample collection

Lung samples were obtained from freshly dead animals, encountered floating or stranded, in the Canary Islands and Andalusia (Spain) between the years 2006–2019 (Table 1). Required permission for the management of stranded cetaceans was issued by the environmental department of the Canary Islands’ Government, the Regional Government of Andalusia, and the Spanish Ministry of Environment. Only fresh dead animals (no bloating nor changes in coloration, edible meat)^46^ were considered for the study as fatty acids start to degrade immediately after death, resulting in the loss of polyunsaturated fatty acids (PUFAs)^47^. Lung samples were obtained during standard necropsies and stored frozen at − 20 °C until the analysis. All tissues were imported into the United States under valid CITES permits.

**Table 1:** Biological and stranding data of the animals included in the present study.

Case number	Species	Age	Body condition	Location	Diving profile	Lung fat emboli	Cause of death
1	*Z. cavirostris*	Subadult	Good	Fuerteventura	Deep	Negative	Others
2	*Z. cavirostris*	Adult	Good	Lanzarote	Deep	Negative	Others
3	*Z. cavirostris*	Adult	Good	Andalusia	Deep	Positive	Gas and fat embolic syndrome
4	*Z. cavirostris*	Adult	Good	Andalusia	Deep	Positive	Gas and fat embolic syndrome
5	*Z. cavirostris*	Subadult	Fair	Andalusia	Deep	Positive	Gas and fat embolic syndrome
6	*P. macrocephalus*	Neonate	–	La Gomera	Deep	Negative	Others
7	*P. macrocephalus*	Calf	Good	Tenerife	Deep	Negative	Others
8	*P. macrocephalus*	Calf	Good	Tenerife	Deep	Positive	Ship strike
9	*P. macrocephalus*	Juvenile	Good	Gran Canaria	Deep	Positive	Ship strike
10	*M. densirostris*	Adult	Very poor	Fuerteventura	Deep	Negative	Others
11	*M. densirostris*	Adult	Poor	Fuerteventura	Deep	Negative	Others
12	*M. densirostris*	Adult	Fair	Fuerteventura	Deep	Negative	Others
13	*G. griseus*	Adult	Good	Fuerteventura	Deep	Negative	Others
14	*G. griseus*	Adult	Fair	Tenerife	Deep	Negative	Others
15	*G. macrorhynchus*	Subadult	Fair	Lanzarote	Deep	Negative	Others
16	*G. macrorhynchus*	Juvenile	Poor	Gran Canaria	Deep	Negative	Others
17	*G. macrorhynchus*	Calf	Poor	Tenerife	Deep	Negative	Others
18	*S. coeruleoalba*	Subadult	Good	Lanzarote	Shallow	Negative	Others
19	*S. coeruleoalba*	Adult	Fair	Tenerife	Shallow	Negative	Others
20	*S. coeruleoalba*	Adult	Poor	Tenerife	Shallow	Negative	Others
21	*S. coeruleoalba*	Subadult	Poor	Tenerife	Shallow	Negative	Others
22	*S. frontalis*	Adult	Fair	Lanzarote	Shallow	Negative	Others
23	*S. frontalis*	Juvenile	Fair	Tenerife	Shallow	Negative	Others
24	*S. frontalis*	Adult	Fair	Tenerife	Shallow	Negative	Others
25	*S. frontalis*	Subadult	Fair	Tenerife	Shallow	Negative	Others

Species were segregated in deep and shallow divers, considering deep divers as those species known to forage at depths deeper than 500 m (*Physeter*, *Ziphius*, *Mesoplodon*, *Globicephala*, and *Grampus*); all others (*Stenella* spp.) were considered shallow divers^48^. To select the animals included in this study, the presence/absence of lung fat emboli was previously evaluated for animals whose pathological findings made them suspect of having lung fat emboli (i.e., those that have been struck by vessels or those presenting a gas embolic syndrome)^18,21^. Lung fat emboli were positive for three *Z. cavirostris* (Cuvier’s beaked whales) presenting gas embolic syndrome, and two *P. macrocephalus* (sperm whales) involved in ship strikes. Control (negative to lung fat emboli) animals were also included for both species (two controls per species) (Table 1). Animals negative for fat embolism from other species were also included in the study to assess lung lipid composition across species and diving profiles: *Mesoplodon densirostris* (Blainville’s beaked whale) (n = 3), *Globicephala macrorhynchus* (short-finned pilot whale) (n = 3), *Grampus griseus* (Risso´s dolphin) (n = 2), *Stenella coeruleoalba* (n = 4), and *Stenella frontalis* (Atlantic spotted dolphin) (n = 4) (Table 1).

### Lipid extraction and analysis

Total lipids were extracted from the lung samples using a modified Folch procedure^49,50^ and reported as percent wet weight (wt%). Three replicates were analysed for each individual.

#### TLC–FID analysis

The main lipid classes in each of the lungs were identified and quantified by thin-layer chromatography with a flame ionization detector (TLC–FID) (Iatroscan MK-6s; Iatron Laboratories, Inc.: Tokyo, Japan). Samples were spotted on chromarods and developed in 94/6/1 hexane/ethyl acetate/ formic acid. Lipid classes were identified through the use of lipid class standards (Nu Chek Prep, Elysian, MN, USA), and quantified as % of total lipid mass (wt%) using a computer software Peak Simple (Peaksimple 3.29, SRI Instruments, Torrance, CA, USA). This analysis provided data on basic lipid classes: triacylglycerols, free fatty acids, cholesterol, and phospholipids. With TLC–FID, wax esters and sterol esters co-elute and thus, cannot be distinguished from one another.

#### HPTLC polar lipids

The lungs of two representative animals of each species (negative to fat embolism) were studied using high-performance thin-layer chromatography (HPTLC).

Polar lipid classes were separated, identified, and quantified using a CAMAG high-performance thin-layer chromatography (HPTLC) system (CAMAG Scientific, Switzerland). Samples were spotted on glass silica gel 60 F_254_ HPTLC plates (Merck 105642, Merck KGaA, Darmstadt, Germany) using a CAMAG ATS 4 autosampler. Standards were obtained from Avanti Polar Lipids (Alabaster, Alabama, USA) and Nu Chek Prep (Elysian, Minnesota, USA). Approximately 10 mg of total lipids were spotted per application. A separation method modified from Vitiello and Zanetta^51^ was used: HPTLC plates were pre-developed in a solvent system of methyl acetate: isopropyl alcohol: chloroform: methanol: 0.25% aqueous KCl (10:10:10:4:3.6 by volume) for 10 cm. Then, spotted plates were developed for 7 cm in the solvent system described above. Any nonpolar lipid classes present in the sample did not migrate in the solvent system used and were therefore not quantified using this technique.

Developed plates were dried in the fume hood for 5 min, dipped in a copper sulfate/sulfuric acid revelation solution (16 g H_3_PO_4_ + 6 g CuSO_4_ + 200 mL distilled H_2_O), and heated at 160 °C for 20 min. Developed plates were then scanned using a CAMAG TLC Scanner 4 (under Tungsten light at 371 nm wavelength) and imaged under RT white light using a CAMAG TLC Visualizer 2. Lipid classes were identified using standards developed on the same plate as the samples and quantified using linear regression of the peak areas of standards.

#### Gas chromatography (GC) analysis

For GC analysis, fatty acids from total lipid extracts were esterified (obtaining total esterified fatty acids from free and esterified fatty acids) and converted to butyl esters (FABEs), rather than methyl esters (FAMEs) to avoid the loss of short-chain components due to their high volatility^52^.

Fatty acids were separated and analysed by GC using a Thermo Trace 1310 GC with a flame ionization detector (FID) in a fused silica column (30 × 0.25 mm internal diameter) (Zebron ZB-FFAP; Phenomenex, Torrance, CA). Helium was used as the carrier gas, and the gas line was equipped with an oxygen and water scrubber. The following temperature program was used to separate fatty acids by carbon chain length: 65 °C for 2 min, then hold at 165 °C for 0.40 min after ramping at 20 °C/min, hold at 215 °C for 6.6 min after ramping at 2 °C/min, and hold at 250 °C for 5 min after ramping at 5 °C/min. Fatty acid peaks were identified based on commercial fatty acids (Nu-Chek Prep, Inc., Elysian, MN) and known samples, in which fatty acids not present in the commercially available standards were identified based upon peak identification performed on a Thermo Trace Ultra GC/Polaris Q MS (Thermo Fisher Scientific, courtesy S. Budge, Dalhousie University) using a similar column. Peak identification was manually confirmed for each run. Peaks were then integrated using appropriate response factors^53^ with the Chromeleon GC software (ver. 7.2.7; Thermo Scientific, Waltham, MA, USA), providing quantification in weight percentage (wt%) for later transformation in mole% based on known molecular weights of all fatty acids present. Unknown fatty acids were assigned the molecular weight of the FABE next to them. Each fatty acid was described using the nomenclature A: BωX, where A is the number of carbon atoms, B is the number of double bonds, and X is the position of the double bond closest to the terminal methyl group.

### Statistical analysis

The software PRIMER, vers. 7 (Plymouth Routines in Multivariate Ecological Research, Primer-E, Ltd., Ivybridge, U.K.), a non-parametric multivariate approach that allows the inclusion of percentage data^54^, and SPSS, vers. 26.0 (SPSS, Inc., Chicago, IL) software were used for statistical analysis. In SPSS, non-parametric statistics were performed due to the small sample size^55^. Among them, the Mann–Whitney U or the Kruskal–Wallis tests for two or more than two independent samples were used. If Kruskal–Wallis test values were statistically significant, post hoc test adjusted to Bonferroni corrections were performed. Statistical significance was set at *p* < 0.05.

Due to the small sample size, the power to detect differences was lower than desired for all the comparison analyses performed in this study.

#### Lipid content

Lipid content data (wt%) was compared among species and between diving profiles in SPSS. Moreover, animals within the species *P. macrocephalus* and *Z. cavirostris* were tested for significant differences due to the presence of fat emboli.

#### Lipid classes and FA signatures

Lipid classes and all fatty acids were first compared between positive and negative animals to fat embolism within the *Z. cavirostris* and *P.macrocephalus* species using SPSS software. As no significant differences were found either for lipid class or fatty acid composition between positive and negative animals in both species, all animals of both species were considered in further statistical analysis.

For PRIMER analysis, only FAs at ≥ 1 mol% in at least one of the lungs analysed (n = 32) were considered for statistical analysis. When a particular fatty acid was not detected in a sample, its concentration was changed from zero to 0.005%. This value was below the minimum detectable level (0.01%), but it was not so small to result in extreme outliers^56^. Resemblance matrices on untransformed data were generated based on Bray–Curtis dissimilarity. Nonmetric multidimensional scaling (MDS, 25 re-starts, Kruskal scheme 1) plots were produced to compare overall lipid profiles of samples. MDS plots placed samples within a two-dimensional space based on the resemblance matrix. As a result, samples that appeared closer together in the two- dimensional space exhibited more similar lipid profiles. A 2D stress value, ranging from 0 to 1, is generated as an output. Low-stress values indicate high reliance on the model, and stress values lower than 0.2 were assumed to indicated confidence in the placement of samples relative to each other^57^. Cluster analysis was used to analyse similarity levels among species.

Analyses of similarities (ANOSIM, one-way, max. permutations = 999) were performed to determine the effect of species and diving profile on lipid class profiles and fatty acid signatures. The null hypothesis of this test is that no differences exist among the groups compared. The global R ranges from 0 to 1, with higher values indicating greater deviation from the null hypothesis. One-way similarity percentages analysis (SIMPER, one-way, based on Bray–Curtis similarity, cut-off percentage = 90) was conducted on all samples, if the analysis of similarities was significant, to determine the lipids classes/fatty acids that contributed the most to the differences observed between groups. Then, lipid classes/fatty acids explaining most of the differences were analysed for significant differences between/among groups in SPSS.

Different fatty acid ratios were calculated considering all fatty acids identified in the lung (n = 98). These were saturated (SFAs) to total unsaturated (MUFAs) (SFAs/(MUFAs + PUFAs)); monounsaturated (MUFAs) and polyunsaturated to total fatty acids (MUFAs/Total FAs and PUFAs/Total FAs); monounsaturated related to polyunsaturated (MUFAs/PUFAs); and the ratio between omega-six and omega-three polyunsaturated FAs (PUFA w6/w3). Ratios between animals positive and negative to fat embolism within the *Z. cavirostris* and *P. macrocephalus* species were compared statistically. As no significant differences were found, all animals of both species were considered in the statistical comparison of ratios among species.

### Evidence of ethical approval

Required permission for the management of stranded cetaceans was issued by the environmental department of the Canary Islands’ Government and the Spanish Ministry of Environment. No experiments were performed on live animals.

## Results

### Lipid content

The mean lipid content of the lung was between 0.91 and 3.43 wt% (Table S1). Although there was some variation, no significant differences in lung lipid content were detected among species (*p* = 0.127) or diving profiles (*p* = 0.601). Besides, no significant differences were detected when comparing positive and negative animals to fat embolism in *P. macrocephalus* (*p* = 0.394) and *Z. cavirostris* (*p* = 0.689).

### Lipid class composition

**Figure 1:**
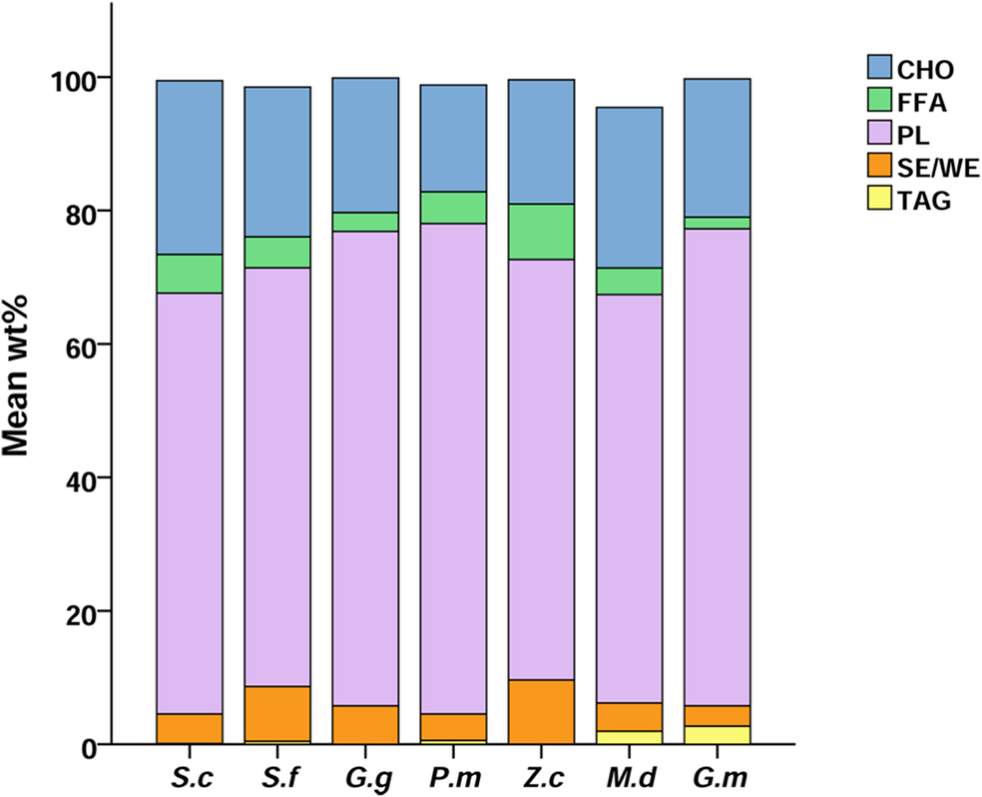
Mean wt% of the lipid classes present in the lung of the seven cetacean species studied. The lipid classes were: CHO = cholesterol, FFA = free fatty acids, PL = phospholipids, SE/WE = sterol esters and TAG = triacylglycerols. The species are: *S.c* = *S. coeruleoalba*, *S.f* = *S. frontalis*, *G.g* = *G. griseus*, *P.m* = *P. macrocephalus*, *Z.c* = *Z. cavirostris*, *M.d* = *M. densirostris*, and *G.m* = *G. macrorhynchus*.

In all lung samples, the lipid classes identified by TLC–FID were (from higher to lower concentrations): phospholipids (50.7–78.5 wt%), cholesterol (10.2–34 wt%), sterol/wax esters (0.8–22 wt%), free fatty acids (0.5–15.2 wt%), and triacylglycerides (0–5.7 wt%) (Table S1, Fig. 1). The wide percentage ranges indicate that there were inter-individual differences in lung lipid classes. In SPSS, non-significant differences in lipid classes were found between the absence and presence of fat emboli in both *Z. cavirostris* and *P. macrocephalus* (all *p* values > 0.05).

Grouping by species, nonpolar lipid percentages among the different species ranged from 25.3 ± 5.2 wt% and 36.6 ± 10.1 wt%, and phospholipids from 61.2 ± 10.6 wt% to 73.5 ± 4.5 wt%. ANOSIM test determined that lipid classes presented non-significant differences among species (*p* = 0.453, global R = − 0.004), and between diving profiles (*p* = 0.232, global R = 0.06).

The most common phospholipids identified by HPTLC in the lung of the animals studied were glycerophosphatides (fatty acid linked to glycerol via an ester bond), and sphingosylphosphatides (fatty acid linked to sphingomyelin via an amide bond). In the first group, phosphatidylcholine (PC) was the most abundant, followed by phosphatidylethanolamine (PE), phosphatidylserine (PS) and phosphatidylinositol (PI) (Table S2). Within the second group, sphingomyelin was also present in high quantities (Table S2).

### Fatty acid composition

Ninety-eight different FA were identified in the lungs, although only thirty-two were present at proportions ≥ 1 mole% in at least one of the lungs analysed. Samples were dominated by fatty acids of at least 16 carbons, with SFAs and MUFAs generally showing the highest percentages (Table 2). The most abundant fatty acids were the SFAs palmitic (C16:0) and stearic (C18:0) fatty acids; the MUFA oleic (C18:1n-9) fatty acid; and the PUFAs arachidonic (C20:4ω6), eicosapentaenoic (20:5ω3), and docosahexaenoic (C22:6ω3) fatty acids. Branched-chain components (< 5.5 mole%) were present in very low proportions. Of these, isolauric acid (*i*-C12:0) was present in the two species from the family *Ziphiidae* in particular (Table 2).

**Table 2:** Fatty acid composition from total lipids (total esterified fatty acids) of the lungs of seven cetacean species.

Mean mole% ± S.D	Z. cavirostris (n = 5)	P. macrocephalus (n = 4)	M. densirostris (n = 3)	G. griseus (n = 2)	G. macrorhynchus (n = 3)	S. coeruleoalba (n = 4)	S. frontalis (n = 4)
C10:0	0.36 ± 0.38	0.02 ± 0.02	0.08 ± 0.10	0.01 ± 0.00	0.02 ± 0.00	0.05 ± 0.04	0.02 ± 0.01
i-C11:0	0.62 ± 0.59	nd	0.03 ± 0.04	nd	nd	0.01 ± 0.01	0.02 ± 0.01
i-C12:0	0.87 ± 0.83	0.00 ± 0.01	0.22 ± 0.28	0.01 ± 0.00	0.00 ± 0.01	0.01 ± 0.01	0.03 ± 0.03
C14:0	0.84 ± 0.18	4.27 ± 2.98	0.96 ± 0.23	1.60 ± 0.68	0.93 ± 0.19	1.26 ± 0.17	1.51 ± 0.26
i-C15:0	0.24 ± 0.18	0.07 ± 0.02	0.29 ± 0.21	1.75 ± 2.04	0.10 ± 0.07	0.62 ± 0.15	0.22 ± 0.14
C15:0	0.20 ± 0.02	0.63 ± 0.26	0.22 ± 0.02	0.86 ± 0.29	0.41 ± 0.02	0.73 ± 0.07	0.93 ± 0.14
i-C16:0	0.08 ± 0.04	0.07 ± 0.03	0.10 ± 0.08	0.62 ± 0.75	0.07 ± 0.04	0.44 ± 0.20	0.18 ± 0.07
C16:0	14.30 ± 2.06	33.91 ± 5.74	14.61 ± 1.17	29.81 ± 0.14	22.29 ± 2.09	23.79 ± 2.64	26.12 ± 4.73
C17:0	0.59 ± 0.13	0.66 ± 0.38	0.54 ± 0.06	0.48 ± 0.67	0.80 ± 0.15	0.67 ± 0.09	1.13 ± 0.19
C18:0	15.18 ± 2.16	10.89 ± 1.73	14.80 ± 1.63	12.11 ± 1.32	13.68 ± 1.34	11.51 ± 1.27	14.52 ± 2.26
**TOTAL SFA**	**35.59 ± 4.51**	**51.41 ± 5.39**	**33.87 ± 1.38**	**49.25 ± 2.79**	**39.79 ± 1.50**	**40.99 ± 3.48**	**46.14 ± 7.44**
C12:1d	0.00 ± 0.01	0.27 ± 0.54	0.11 ± 0.19	0.12 ± 0.16	nd	nd	nd
C16:1ω9	1.66 ± 0.63	1.79 ± 0.41	0.81 ± 0.16	1.12 ± 0.48	0.59 ± 0.33	1.08 ± 0.10	0.27 ± 0.19
C16:1ω7	2.78 ± 0.90	3.70 ± 3.89	2.87 ± 0.18	3.20 ± 2.93	2.70 ± 1.66	3.04 ± 0.84	2.10 ± 0.75
C18:1ω11	0.39 ± 0.23	0.35 ± 0.25	1.87 ± 0.70	nd	0.23 ± 0.38	0.34 ± 0.13	0.02 ± 0.04
C18:1ω9	25.72 ± 4.11	17.47 ± 2.81	29.20 ± 2.43	18.00 ± 2.57	23.09 ± 2.79	16.42 ± 0.76	15.74 ± 1.85
C18:1ω7	2.29 ± 1.79	1.40 ± 0.78	3.01 ± 0.23	2.49 ± 0.95	2.86 ± 0.32	2.11 ± 0.25	2.45 ± 0.67
C20:1ω11	0.73 ± 0.33	0.19 ± 0.16	1.30 ± 0.40	0.26 ± 0.05	0.56 ± 0.30	0.30 ± 0.09	0.07 ± 0.05
C20:1ω9	2.69 ± 0.88	1.16 ± 0.65	2.33 ± 0.21	1.55 ± 0.20	2.82 ± 0.44	2.72 ± 0.87	1.05 ± 0.49
C22:1ω11	0.66 ± 0.46	0.13 ± 0.09	0.82 ± 0.33	0.09 ± 0.00	0.31 ± 0.18	0.35 ± 0.13	0.06 ± 0.04
C24:1ω11	1.32 ± 0.78	0.74 ± 1.45	1.11 ± 0.79	0.76 ± 0.14	1.84 ± 0.24	2.87 ± 2.55	4.20 ± 4.27
C24:1ω9	0.87 ± 0.64	0.67 ± 0.09	1.32 ± 0.11	1.61 ± 0.69	1.27 ± 0.30	1.65 ± 0.10	1.11 ± 0.42
**TOTAL MUFA**	**41.19 ± 6.69**	**29.29 ± 5.02**	**46.21 ± 1.75**	**30.75 ± 8.36**	**37.78 ± 3.05**	**32.32 ± 3.36**	**28.34 ± 3.19**
C16:2ω4	1.11 ± 0.61	1.60 ± 0.69	1.36 ± 0.74	1.08 ± 0.15	0.55 ± 0.04	0.65 ± 0.41	0.89 ± 0.45
C16:4ω1	0.27 ± 0.60	nd	0.32 ± 0.55	nd	nd	0.02 ± 0.02	nd
C20:2ω6	0.22 ± 0.11	0.23 ± 0.19	0.15 ± 0.13	0.11 ± 0.01	0.35 ± 0.15	0.51 ± 0.27	0.80 ± 0.63
C20:4ω6	5.72 ± 3.34	5.82 ± 1.67	5.24 ± 4.63	4.17 ± 5.89	9.98 ± 1.18	8.17 ± 1.05	5.20 ± 3.98
C20:5ω3	5.26 ± 2.39	3.25 ± 1.46	2.54 ± 0.51	4.03 ± 2.73	2.98 ± 0.48	4.34 ± 0.69	2.68 ± 1.35
C22:4ω6	0.37 ± 0.21	0.66 ± 0.16	0.59 ± 0.10	1.42 ± 0.29	1.17 ± 0.30	1.03 ± 0.19	1.23 ± 0.50
C22:5ω3	1.55 ± 0.36	1.40 ± 0.36	1.14 ± 0.23	2.18 ± 0.67	1.73 ± 0.31	2.11 ± 0.36	1.89 ± 0.48
C22:6ω3	3.24 ± 1.91	3.18 ± 1.34	2.63 ± 0.46	4.18 ± 1.74	3.17 ± 0.19	3.64 ± 0.56	5.04 ± 1.96
**TOTAL PUFA**	**19.74 ± 4.58**	**17.91 ± 4.59**	**16.31 ± 5.21**	**18.57 ± 10.42**	**21.28 ± 2.39**	**22.27 ± 2.49**	**20.09 ± 6.73**
Unknown FA2	1.42 ± 2.25	0.03 ± 0.06	1.30 ± 2.24	nd	nd	1.00 ± 0.91	1.39 ± 1.63
Unknown FA3	0.43 ± 0.42	0.15 ± 0.11	0.77 ± 0.66	0.56 ± 0.26	0.00 ± 0.01	0.28 ± 0.29	0.24 ± 0.29
Unknown FA5	1.28 ± 0.76	0.60 ± 1.09	1.08 ± 0.92	0.63 ± 0.29	0.94 ± 0.15	2.64 ± 2.33	3.40 ± 3.38
**TOTAL UNKNOWN FA**	**3.48 ± 2.79**	**1.39 ± 1.27**	**3.03 ± 2.28**	**1.43 ± 0.73**	**1.16 ± 0.15**	**4.43 ± 2.70**	**5.44 ± 4.09**
**SFA/(MUFA + PUFA)**	**0.59 ± 0.12**	**1.10 ± 0.20**	**0.54 ± 0.06**	**1.00 ± 0.10**	**0.75 ± 0.09**	**0.98 ± 0.28**	**0.68 ± 0.04**
**MUFA/TOTAL FA**	**0.41 ± 0.07**	**0.29 ± 0.05**	**0.46 ± 0.02**	**0.31 ± 0.08**	**0.32 ± 0.03**	**0.28 ± 0.03**	**0.38 ± 0.03**
**PUFA/TOTAL FA**	**0.20 ± 0.05**	**0.18 ± 0.05**	**0.16 ± 0.05**	**0.19 ± 0.10**	**0.22 ± 0.03**	**0.20 ± 0.07**	**0.21 ± 0.02**
**MUFA/PUFA**	**2.24 ± 0.86**	**1.75 ± 0.61**	**3.11 ± 1.31**	**2.12 ± 1.64**	**1.48 ± 0.30**	**1.56 ± 0.62**	**1.80 ± 0.32**
**PUFA n-6/n-3**	**0.78 ± 0.56**	**0.94 ± 0.17**	**1.04 ± 0.61**	**0.54 ± 0.25**	**1.01 ± 0.11**	**0.88 ± 0.24**	**1.49 ± 0.05**

Fatty acid values are given in mean mole% ± S.D. Only fatty acids that showed a proportion of over 1 mole% in at least one of the animals were included in the table (n = 32). Values of total saturated (total SFA), monounsaturated (total MUFA), polyunsaturated (total PUFA), unknown (totally unknown) fatty acids, and ratios are shown as well (in bold) and were calculated from the total fatty acids (n = 98) identified in the lung. As there were no significant differences in the fatty acid composition of animals positive/negative to fat emboli, all animals from these two species were considered in the table (*Z. cavirostris* n = 5 and *P. macrocephalus* n = 4).

Differences in fatty acid composition between absence and presence of fat emboli in both *Z. cavirostris* and *P. macrocephalus* were not significant (all *p* values > 0.05)*.*

**Figure 2:**
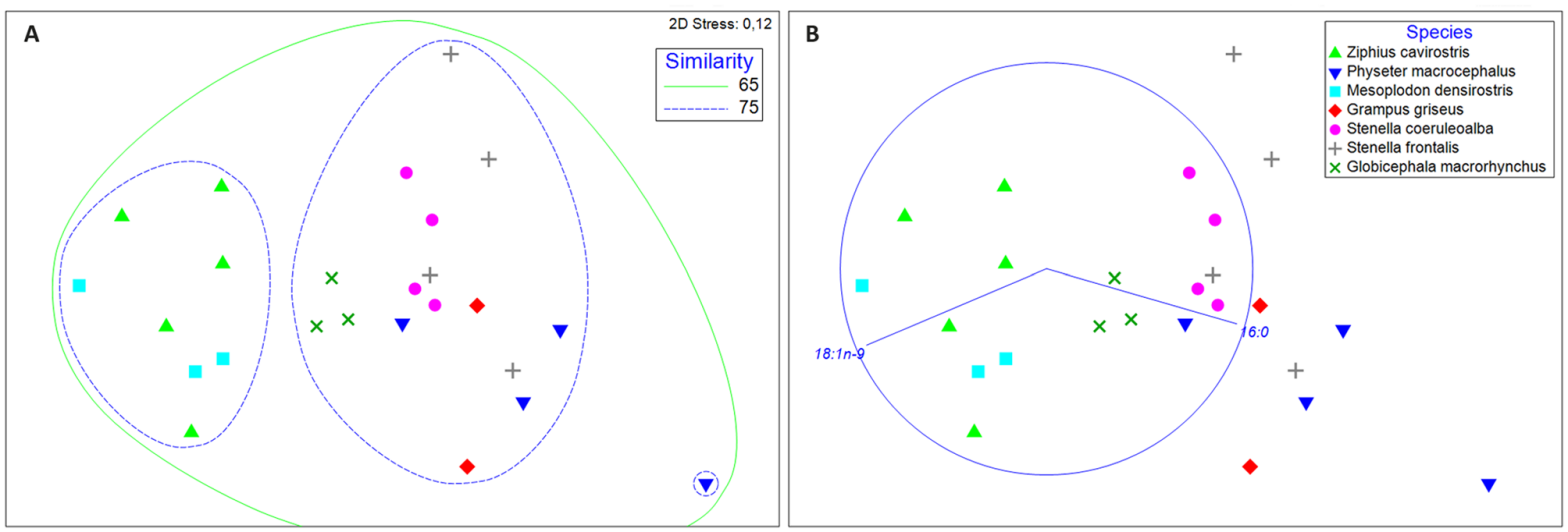
nMDS plot (Euclidean distance) of untransformed fatty acid proportions (n = 32) in the lungs of the animals studied. They are separated by species, with a different symbol and colour, and plotted individually based on the similarity of fatty acid signature. (**A**) Contours are a result of cluster analysis and represent 65% and 75% similarity. (**B**) Vectors indicate the top two FAs identified by SIMPER analyses that distinguish FA profiles among species.

The nMDS plot 2D stress value was 0.12 (< 0.2), indicating that the model was confident in the placement of the points (Fig. 2). The ANOSIM revealed non-significant differences in the fatty acid composition between diving profiles (*p* = 0.08; R = 0.149) but between species (*p* = 0.001, global R = 0.526).

The cluster analysis revealed that the lung fatty acid composition of the different species studied was over 65% similar. All the individuals from the family *Ziphiidae* were enclosed in a cluster revealing a fatty acid composition similarity of over 75% (Fig. 2A). The rest of the species were enclosed in another 75%-similarity cluster except for a *P. macrocephalus* neonate, found in a separate 75% similarity cluster (Fig. 2A).

SIMPER analyses revealed that the three fatty acids that contributed the most to the similarity within a single species were: C16:0 (17.1–39.1 mol%), C18:1ω9 (19.1–34.2 mol%), and C18:0 (12.5–18.3 mol%), being their cumulative effects between 58.4 and 74.8 mol%. At the same time, C16:0 and C18:1ω9, were the most influential in separating the fatty acid signatures among species. They were always among the top contributors to the dissimilarities between species (C16:0 (5.5–32.9%) and C18:1ω9 (4.5–26.2%)) with a cumulative difference effect between 16.6 and 50.0% (Fig. 2B). Significant differences were found among species (*p* < 0.001), as individuals from the Family *Ziphiidae* had higher levels of C18:1ω9 and lower values of C16:0 than the other groups (Fig. 2B).

The statistical comparison of ratios revealed non-significant differences between animals positive and negative to fat embolism for *Z. cavirostris* and *P. macrocephalus*, which was expected as non-significant differences were observed between fatty acids related to fat emboli. In the comparison among the seven species, significant differences were found between *S. frontalis* and *M. densirostris* in the MUFAs/Total FA ratio (*p* = 0.039); and between *P.macrocephalus* with both *Z. cavirostris* (*p* = 0.036) and M. densirostris (*p* = 0.039) for FAs/Total FAs ratios.

The chromatogram analysis revealed the presence of an unknown fatty acid (Unknown FA1) at 5.96 min in the lungs of the two *P. macrocephalus* positive to fat embolism, but it was not present in the individuals negative to fat embolism or in the other species analysed. The percentage concentrations of this fatty acid were 0.6 mol% (Case 8) and 0.8 mol% (Case 9) (Fig. S1). The structure of this fatty acid is currently unknown.

## Discussion

### Lipid content

Lipid content showed intra-individual variability and was not associated with the diving profile or the presence of lung fat embolism. Lung lipid content ranged between 1 and 2% in all the species studied. These values agree with previous studies in striped dolphins^35,36,58^, and other species, such as humans^59^. A study carried out by Storelli and colleagues in 1999, reported lower lipid percentages in the lung of a Cuvier´s beaked whale and a Risso’s dolphin (0.4 and 0.66%, respectively). Nevertheless, it should be highlighted that these previous values are based on just one animal of each of these two species, and the condition of the carcasses in this study was not reported^58^.

### Lipid classes composition

Lipid classes in lung tissue did not differ between positive and negative for fat embolism, or among species. Lipid classes present in cetacean lungs were the same as those previously reported in the literature for various species, as in the study published by Clements about the lipid composition of 11 terrestrial and aquatic vertebrate species^60^. Moreover, amounts of nonpolar lipids (cholesterol, cholesterol ester, FFA, and TAG) and phospholipids in the lungs of the cetacean species studied here were very similar to those described in other species such as dogs^61^, rats^62^, and humans^63^.

Among polar lipid classes, most studies have focused on lung surfactant composition, which is a material produced and secreted by the lung epithelium to assist in alveolar stability. Lung surfactant is composed of phospholipids (90%), specifically phosphatidylcholine, and proteins (10%)^45^. Phospholipids present in the surfactant are also present in the lung tissue, as part of lamellar bodies, although their proportions are different (in lavage fluid: 75–90% and in lamellar bodies: 60%)^43^. For example, phosphatidylcholine is the dominant phospholipid class in both surfactant and lung tissue, but its content is higher in the surfactant than in the lung tissue. As well, phosphatidylglycerol is the second most abundant phospholipid in the surfactant, but its contribution is much lower in the lung tissue. In contrast, phosphatidylethanolamine is abundant in the lung tissue but is present in low amounts in the surfactant^43^.

Although we do not know how much of the polar lipids identified in the lungs of the animals in the current study were from surfactant or for the lung tissue itself, phosphatidylcholine and phosphatidylethanolamine constituted the major phospholipid classes identified, as it is the case for lung tissue. Other phospholipids also present in substantial amounts were sphingomyelin, phosphatidylserine, and finally, phosphatidylinositol, being this constancy kept among several species such as dogs, humans, chickens or bovines among others^64^.

### Fatty acid composition

The fatty acids identified in the lungs were generally similar among the different species studied, with only a few of them presenting significant quantitative differences. In all the species, except the two species from the family *Ziphiidae*, SFAs showed the highest percentage, followed by MUFAs and finally PUFAs, in agreement with previous studies in striped dolphins^36,37^.

The most abundant fatty acids in the lung of all the animals studied were those commonly encountered in most animal tissues. These were the SFAs palmitic (C16:0) and stearic (C18:0) acids, the MUFA oleic acid (C18:1ω9), and the PUFAs arachidonic (C20:4ω6), eicosapentaenoic (C20:5ω3), and docosahexaenoic (C22:6ω3)^1,6,65^. Among the identified fatty acids, palmitic and oleic acids showed the highest proportion in all the lungs. The same results were observed in previous studies of the lipid composition of the lung in several vertebrate species, including sea lions, marine turtles, and humans^37,60^.

Although the most prevalent fatty acids present in the lung seemed to be conserved among species, there were significant differences in the fatty acid profile among species. *M. densirostris* and *Z. cavirostris* (family *Ziphiidae*) showed a higher amount of the monoene oleic acid and a lower amount of the saturated palmitic acid compared to the other species studied. These differences explain the grouping of these two species in a separate cluster from the other species, including some deep-diving species, and their differences in SFAs/Total FAs and MUFAs/Total FAs compared to the rest of species. For our purposes, with the species in this study, we cannot separate the effects of the diving regime from those of taxonomy; this question deserves further attention.

Other fatty acids present in the lungs of the various species described by Clements^60^, such as myristic acid (C14:0) or the C16:1 group, were also present in our animals in similar amounts. The C18:2 group was present in the lung of most of the vertebrates studied by Clements, being the sea lion the species showing the lowest percent (2.5 molar percent)^60^. Similarly, in all the cetacean species included in this study, the percentages of this group were very low (0.35–0.86 mean mole%).

PUFAs constitute essential fatty acids in mammals, which means that they have to be obtained through the diet as mammals lack the needed desaturase enzymes to synthesize them^66^. Among them, arachidonic, eicosapentaenoic, and docosahexaenoic acids are present in higher amounts in tissues, as they constitute major components of membrane phospholipids throughout the animal kingdom. Arachidonic acid is the PUFA showing the highest content in the lungs of all the seven cetacean species included in the study. This finding agrees with previous studies in striped dolphins that concluded that this fatty acid was the most important not only in lungs but in all the other tissues studied, including melon, cerebrum, liver, or muscle^36,37,67^. The high ω6:ω3 ratio (≈1:1) in the lungs of the toothed whale species included in this study has also been described in different tissues of *S. coeruleoalba*, being more similar to land mammals than to other marine species, where the ratio is lower (as marine lipids are rich in ω3 fatty acids^1^). Williams and colleagues have suggested that this could indicate that marine mammals have maintained the requirements of the ω6-fatty acid from their terrestrial ancestors^67^.

Other fatty acids present in the lungs of the various toothed-whale species studied were branched-chain fatty acids (BCFA). They are endogenous fatty acids, derived from the catabolism of branched-chain amino acids^68^. Among them, short branched-chain fatty acids appear in high concentrations in the acoustic fats and blubber of some species of toothed whales^3,6,9^. Some of the most important ones are isovaleric acid (*i-*5:0) in *Delphinidae*^2,3,6,9,13,34^, *Phocoenidae*^2,3^, and *Monodentidae*^69^ families; and isolauric acid (*i*-C12:0) in the acoustic fats of the *Ziphiidae* family. In delphinids, *i*-C5:0 has been detected, but in much lower quantities, in other tissues, such as liver and muscle, indicating a certain degree of synthesis and deposition of this BCFA in those tissues^2^. Despite its presence in various cetacean tissues, *i*-C5:0 was not present in the lungs of the different delphinid species included in the present study, in agreement with previous research in an adult porpoise^38^.

In ziphiids, *i*-C12:0 constitutes a major fatty acid in melon and mandibular fat tissues^3,4,7,13^, and it was also present, although in much lower quantities, in their lung tissue. In those species in which *i*-C12:0 was not detected in acoustic tissues, such as *P. macrocephalus* and delphinid species^4^,* i*-C12:0 was present in negligible quantities (close to 0) or not present at all in the lung.

Considering all that was mentioned previously, fatty acids present in the lungs seem to be phylogenetically conserved across toothed whale species, with only small differences in the presence (*i*-C12:0) and proportion of specific fatty acids (C16:0 and C18:1ω9). This fact differs from other toothed whale tissues, like acoustic fats, where a robust phylogenetic diversity in the fatty acids present has been described^4^.

Concerning fat embolism, differences in fatty acid composition or the presence of fatty acids characteristic from other tissues in the lungs of *P. macrocephalus* and *Z. cavirostris* positive to fat embolism, could not be established in this study. The only evidence of a potential signal of fat embolism was the presence of an unknown short-chain fatty acid at the beginning of the chromatogram of the two ship-strike *P. macrocephalus* (Fig. S1). Further research should be carried out to identify this fatty acid and see if a relationship with the fatty acid composition of the marrow from the fractured bone or the injured soft tissue of the struck animals can be established.

We suggest that future research should be focused on the identification of fatty alcohols in the lungs of beaked whales presenting a gas embolic pathology. Since wax esters consist of a fatty alcohol esterified to a fatty acid, the presence of fatty alcohols indicates that component was sourced exclusively from a wax ester. Wax esters are the major components in both acoustic fats and the blubber of species from the family *Ziphiidae*^3,4^, and nitrogen has proven to be highly soluble in them^33,34^. Thus, the presence and identification of fatty alcohols exclusively in the lungs positive to fat embolism would provide invaluable knowledge to the etiopathogenesis of fat embolism in these animals. As sperm whales´ blubber is mostly composed of waxes^10^, and this tissue, together with muscles and bones, is one of the most affected when a ship-strike occurs^19,20^, identification of fatty alcohols in the lungs of struck animals would be of great interest, as well.

To conclude, lipid content and lipid profiles (lipid classes and fatty acids identified) seem to be conserved among lung tissue in the animal kingdom, and variability in fatty acid amounts seem to be driving the differences among species in this tissue, e.g., oleic acid in beaked whales. The knowledge provided in this study on regular lung tissue lipid composition in cetaceans will be extremely useful in future studies aiming to assess lung pathologies involving lipids.

## Supplementary information

Supplementary information.

## Data Availability

All the samples reported in this work are stored in the tissue bank of the Institute of Animal Health and Food Safety (IUSA). Veterinary School. University of Las Palmas de Gran Canaria.
