# Supplementary material for: Lipids of lung and lung fat emboli of the toothed whales (Odontoceti)
Source: Sci Rep. 2020 Sep 8;10:14752. doi: 10.1038/s41598-020-71658-8 (PMC7479150; doi:10.1038/s41598-020-71658-8)
Supplement: Supplementary file 1 — Supplementary information. [file 41598_2020_71658_MOESM1_ESM.docx]

**Lipids of lung and lung fat emboli of the toothed whales (Odontoceti)**

**Marina Arregui^1^, Hillary Lane Glandon^2^, Yara Bernaldo de Quirós^1*^, Idaira Felipe-Jiménez^1^, Francesco Consoli^1,3^, María José Caballero^1^, Heather N. Koopman^2^, and Antonio Fernández^1^**

1. **Supplementary information**

Table S1. Percentage of lipid content and lipid class composition (wt%) of the lung of the different species studied. Values given are the mean percent content of total lipid ± S.D. As there were no significant differences in the fatty acid composition of animals positive/negative to fat emboli, all animals from these two species were considered in the table (*Z. cavirostris* n=5 and *P. macrocephalus* n=4)

| Species | Lipid content | taG | ffa | Sterol Ester | cholesterol | Phospholipids |
| --- | --- | --- | --- | --- | --- | --- |
|  | **Mean wt% ± S.D.** | | | | | |
| *Z. cavirostris* | 2.33 ± 0.68 | 0.01 ± 0.03 | 8.32 ± 6.22 | 9.61 ± 7.45 | 18.61 ± 7.40 | 63.03 ± 10.45 |
| *P. macrocephalus* | 1.75 ± 0.20 | 0.57 ± 0.76 | 4.76 ± 2.34 | 3.97 ± 4.64 | 16.01 ± 1.02 | 73.50 ± 4.54 |
| *M. densirostris* | 1.55 ± 0.20 | 1.95 ± 1.18 | 3.99 ± 0.45 | 4.24 ± 3.83 | 24.07 ± 3.35 | 61.20 ± 10.60 |
| *G. griseus* | 1.43 ± 0.33 | 0.00 ± 0.00 | 2.81 ± 3.30 | 5.76 ± 1.42 | 20.18 ± 3.92 | 71.10 ± 8.70 |
| *G. macrorhynchus* | 1.42 ± 0.21 | 2.72 ± 2.62 | 1.75 ± 0.54 | 3.04 ± 0.72 | 20.71 ± 1.03 | 71.49 ± 3.92 |
| *S. coeruleoalba* | 1.66 ± 0.40 | 0.11 ± 0.15 | 5.79 ± 1.96 | 4.44 ± 1.43 | 26.05 ± 8.98 | 63.07 ± 8.46 |
| *S. frontalis* | 1.51 ± 0.54 | 0.45 ± 0.41 | 4.66 ± 4.45 | 8.21 ± 3.44 | 22.42 ± 8.08 | 62.74 ± 8.66 |

Table S2. Concentration (ug/mL) of the most important phospholipid classes identified in the lungs of the samples analysed. PC= phosphatidylcholine, PE= phosphatidylethanolamine, PI= phosphatidylinositol, PS= phosphatidylserine, Sphm= sphingomyelin.

| **Species** | **Case** | **PC** | **PE** | **PI** | **PS** | **Sphm** |
| --- | --- | --- | --- | --- | --- | --- |
|  |  | **Concentration (ug/mL)** | | | | |
| ***Z. cavirostris*** | 1 | 738.5 | 520 | 346.2 | 537.2 | 483 |
|  | 2 | 288.7 | 276.2 | 70.26 | 132.9 | 221.5 |
| ***P. macrocephalus*** | 6 | 681.3 | 458.9 | 435 | 499.8 | 313.7 |
|  | 7 | 699.1 | 465.7 | 445 | 349.2 | 268.9 |
| ***M. densirostris*** | 11 | 695.2 | 501 | 160.4 | 335 | 502.4 |
|  | 12 | 596.9 | 466.2 | 106.6 | 252.2 | 444.6 |
| ***G. griseus*** | 13 | 869.5 | 552.3 | 388.7 | 570.7 | 482.9 |
|  | 14 | 468.4 | 299.4 | 100.1 | 283.7 | 415.9 |
| ***G. macrorhynchus*** | 15 | 759.2 | 514.9 | 257.4 | 380.5 | 525.4 |
|  | 17 | 895 | 502.9 | 346.7 | 417.1 | 515.5 |
| ***S. coeruleoalba*** | 18 | 399.4 | 307.5 | 116.9 | 176.5 | 310.4 |
|  | 21 | 531.6 | 350.8 | 182.6 | 237.6 | 290.7 |
| ***S. frontalis*** | 22 | 869.6 | 471.2 | 407.4 | 289.2 | 497.9 |
|  | 25 | 807.6 | 416.3 | 307.8 | 260.6 | 487.5 |


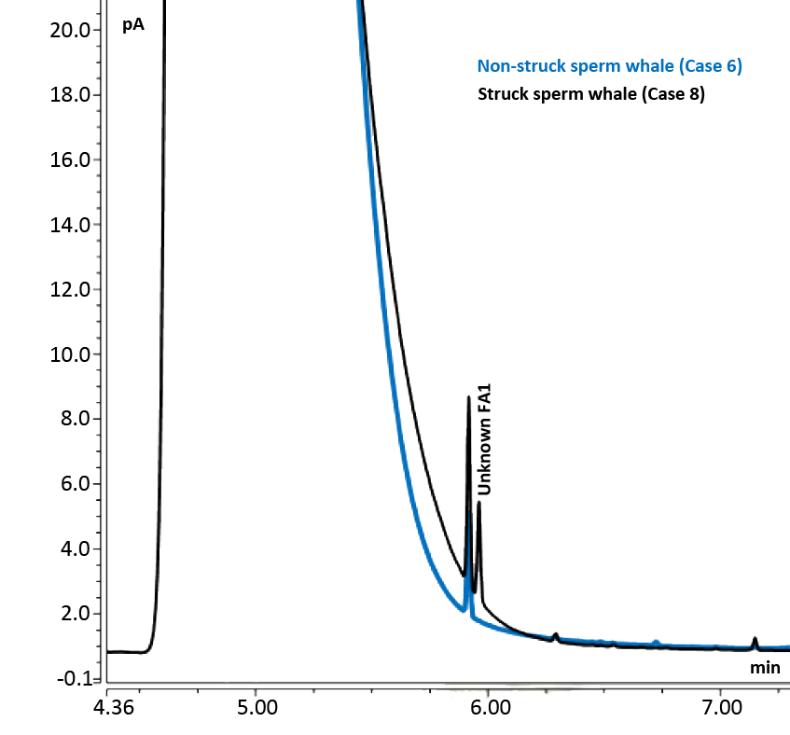


**Fig. S1. Chromatogram inset of one struck sperm whale (case 8) and one non-struck sperm whale (case 6).** At 5.96 min, presence of an unknown fatty acid (Labelled as “Unknown FA1”) in the chromatogram of the struck sperm whale but not in the non-struck sperm whale.
